# Supplementary material for: Examining health sector stakeholder perceptions on the efficiency of county health systems in Kenya
Source: PLOS Glob Public Health. 2021 Dec 23;1(12):e0000077. doi: 10.1371/journal.pgph.0000077 (PMC10021822; doi:10.1371/journal.pgph.0000077)
Supplement: S1 Appendix — (DOCX) [file pgph.0000077.s001.docx]

**S1 Appendix: TOPIC guides for Focus Group Discussions**

**Examining Health Sector Stakeholder Perceptions on the Efficiency of County Health Systems in Kenya**

1. Could you describe what your understanding of county health system efficiency
2. In your opinion, what inputs should be considered are relevant when assessing the efficiency of county health systems
3. In your opinion, what outputs should be considered are relevant when assessing the efficiency of county health systems
4. In your experience, what factors affect the efficiency of county health systems in Kenya?
5. How do these factors influence the efficiency of county health systems? Could you illustrate with an example?
