# Supplementary material for: Examining health sector stakeholder perceptions on the efficiency of county health systems in Kenya
Source: PLOS Glob Public Health. 2021 Dec 23;1(12):e0000077. doi: 10.1371/journal.pgph.0000077 (PMC10021822; doi:10.1371/journal.pgph.0000077)
Supplement: Attachment — Submitted filename: Response to reviewer comments.docx [file pgph.0000077.s002.docx]

Examining Health Sector Stakeholder Perceptions on the Efficiency of County Health Systems in Kenya

**Responses to Reviewer’s Comments**

| **Comment** | **Authors Response** |
| --- | --- |
| **Reviewer 1** |  |
| **Introduction** |  |
| After line 56, create a paragraph briefly describing the health system structure & present health financing arrangements for Kenya. Also, include some of the government strategies for realization of UHC & progress towards those UHC goals for Kenya. | We have now included information on the Kenyan health system structure, and financing arrangements, as well as UHC plans in several sections of the introduction |
| Line 58: Preferably, change 'Efficiency' to 'Health system efficiency and 'system' to 'health system'. | This suggestion has been implemented in the revised manuscript |
| Lines 65-67, pg3, while structurally devolution led to two tiers of health system governance, kindly explain briefly the functional responsibilities of national & county health systems after devolution here. | We have now provided an explanation of the functional responsibilities of the national vs county governments |
| Line 81-84: Kindly provide a reference explaining that tacit knowledge can be expressed through verbalization (FGD or interview as data collection tools) or writing (narratives). I would humbly suggest to replace 'tacit knowledge' with 'knowhow'. Kindly check the following books & one article if needed: 1. Polkinghorne, D. (2004). Practice and the human sciences: The case for a judgment-based practice of care. Suny Press. (pg 2-5) 2. Polanyi, M. The Tacit Dimension. 1966 Chicago. 3. Visser, F. S., Stappers, P. J., Van der Lugt, R., & Sanders, E. B. (2005). Contextmapping: experiences from practice. CoDesign, 1(2), 119-149. | We have now replaced tacit knowledge with knowhow as suggested by the reviewer |
| Line 84: Preferably change 'empirical analysis' to 'quantitative assessment' here. | This suggestion has been implemented in the revised manuscript |
| Methods: |  |
| Study Design & Data Collection: |  |
| 1. What is the study design and methodology (not just the data collection method)? Why such an approach has been selected to answer the research questions? (kindly use content from lines 533-549 here for the purpose) | We have now revised the methods section to include a description of the study design. We have also moved the justification for a qualitative approach from the discussion section to the methods section |
| 2. What is your ontological & epistemological stance for this research? Some references which can be used:  (a) Giorgi, A. (2020). Reflections on certain qualitative and phenomenological psychological methods. University Professors Press. (b)Holloway, I. (1997). Basic concepts for qualitative research. Wiley-Blackwell. | We have now provided information on our ontological and epistemological stance |
| 3. How did you attempt to ensure trustworthiness & rigour for your study? Preferably, use the below stated reference for criteria to answer this point: Lincoln, Y. S., & Guba, E. G. (1986). But is it rigorous? Trustworthiness and authenticity in naturalistic evaluation. New directions for program evaluation, 1986(30), 73-84. | We have now included information on measure to ensure rigor and trustworthiness |
| 4. Why did you choose Focus Group Discussion (FGD) for data collection? | We have now provided this justification in the methods section |
| 5. Why specifically is the term 'workshop' being used instead of simply 'focus group discussion'? | We have now clarified that we carried out FGDs during the workshop |
|  |  |
| Data Analysis: |  |
| Kindly break down the term 'we' here to show responsibilities of various researchers during this data analysis stage like you have explained for data collection in lines 117-119. This will ensure further transparency and enhance rigour. Further, any data triangulation, member checking, peer debriefing performed during or after data analysis? | We have now provided details on who carried out specific responsibilities. We have also clarified that we carried out member checking, and peer debriefing |
| Line 99-100: Kindly consider replacing these lines to "For this study, we adopted purposive sampling of participants to gain diverse perspectives from respondents in order to investigate our research objectives". | This suggestion has been implemented in the revised manuscript |
| Kindly attach a copy of the group discussion topic guide as an annexure for this paper. | We have now included the topic guide as a supplementary file |
| Line 124: Probably add a line: "We repeatedly listened to the audio recordings and checked with the content of the transcripts prior to data analysis". | This suggestion has been implemented in the revised manuscript |
| Line 128: Modify "insights from the data" to preferably "insights from the anonymised transcribed data". | This suggestion has been implemented in the revised manuscript |
| Ethics: |  |
| Mention here how data privacy & confidentiality was ensured. | We have now outlined how we maintained privacy and confidentiality |
| Results: |  |
| Kindly start with a brief description of how your results section is laid out with a synopsis of the details of the expected themes that you are going to inform further. | This suggestion has been implemented in the revised manuscript |
| Stakeholder Understanding of the Efficiency of County Health Systems: |  |
| Line 143: Kindly explain in 2-3 lines what is meant by health system inputs, outputs and outcome here for the convenience of the readers. | We have now provided an explanation of health system inputs |
| Line 145: Preferably modify to : "Participants highlighted that efficient healthcare delivery should be cost effective and responsive to community healthcare needs. Therefore, a system wide approach might be needed for improving health system efficiency." Preferably place a line in between line no 157-158: "In some cases, the formal county health authority may not have practical authority for health system resource allocation & distribution in the country." | This suggestion has been implemented in the revised manuscript |
| Relevant Inputs to the County Health System: |  |
| Line 172: Explain the relevance or importance of health system inputs in regulating health system efficiency here in 2-3 lines. | We have now included an explanation of the relevance of health system inputs |
| Line 173: Preferably modify : "Hard inputs were consistent with health system building blocks" to "Hard inputs were consistent with the health system strengthening (HSS) components". | This suggestion has been implemented in the revised manuscript |
| Line 174: May be add health service delivery and health system governance explicitly here. | This suggestion has been implemented in the revised manuscript |
| Line 175: Change "such" to "such as" | This suggestion has been implemented in the revised manuscript |
| Lines 175-176: Improve the lines with the following additions: policies & guidelines for? managerial system for? | This suggestion has been implemented in the revised manuscript |
| Between line 176-177 considering adding a line: " Participants highlighted the need for emphasis on soft inputs and also expressed the difficulty in assessing them to ascertain the health system efficiency." | This suggestion has been implemented in the revised manuscript |
| Line 187: Consider replacing, "optimal mix of inputs for county" to "optimal mix of inputs especially healthcare commodities for county..." | We appreciate this comment. However, we would prefer to leave it as it is, since it would deviate from our intended meaning |
| Line 188: Preferably, change 'inputs' to 'health system strengthening components'. | This suggestion has been implemented in the revised manuscript |
| Relevant Outputs of the County Health System Kindly start with explaining the relevance or importance of health system output in regulating health system efficiency here in 2-3 lines. | We have now provided an explanation of the importance of health system outputs |
| Line 206-207: Preferably modify to: "Respondents felt that health output & outcome indicators used in quantitative assessment of county health system efficiency should represent the disease burden of the county." | This suggestion has been implemented in the revised manuscript |
| Line 207-209: Preferably modify to: "They also highlighted the need to select standardized output & outcome indicators that could be implemented nationally across all counties of Kenya for facilitating valid comparisons." | This suggestion has been implemented in the revised manuscript |
| Lines 209-213: Consider modifying to :"Participants highlighted the dual burden of disease in Kenya, with an increasing burden of NCDs along with a continued relevance for infectious diseases & RMNCAH. Therefore, respondents expressed the need for monitoring of intervention coverage and health outcome indicators relevant for both NCDs and infectious diseases which could be compared across 47 counties." | This suggestion has been implemented in the revised manuscript |
| Line 224: consider changing "...the importance of including the quality of care provided by the county health system as an output." to "... the relevance of including quality of care provided at the healthcare institutions as an important dimension for assessing the health system efficiency." | This suggestion has been implemented in the revised manuscript |
| Factors Affecting County Health System Efficiency |  |
| Public Finance Management |  |
| Lines 236-238: Is the disbursement issue between national & county level or from county to facility level? The excerpts from lines: 241-243: "Money was received by counties by 20th December [2018] and three to four months down the line, money was still not at facility level and these are facilities that are not collecting user fees." suggest county to facility level?  This is important as huge amount of health finances (roughly i guess 60%) goes to the county level in Kenya. Is there any content from the transcript to dive deeper into the issue of why there is delay in disbursement? If not, then kindly show it a scope for future research later in the conclusion section of the paper. | We have now clarified that the disbursement issue is both between the national level and the county level, and the county level and the healthcare facilities  We do not have additional quotes to provide further details on this issue. We have flagged it as an area for further research in the discussion section |
| Line 248: Preferably modify: "...autonomy of health facilities" to "autonomy of health facilities and the need to assess facility level efficiency". ( taken from lines 265-266) | We appreciate this comment. We would prefer to leave I as it is to avoid deviating from our intended meaning |
| Lines 249-251: Please mention pathways in which funds are collected by public health facilities (only user fee or other options as well?). | We have now provided information on the sources of funding for health facilities |
| Human Resources for Health |  |
| Preferably use the term "Health Professionals" or "Healthcare Staff" than "health workers" in appropriate places (where you talk about specialty doctors etc.). | This suggestion has been implemented in the revised manuscript |
| Lines 273-275: Preferably modify to: "For example, participants felt that the efficiency of county health system was affected by high workload and inadequate number of health workers employed by counties. " (info taken from lines 281-284) | This suggestion has been implemented in the revised manuscript |
| Line 286: Change 'mix' to 'skill mix' and 'health workers' to 'health professionals'. | This suggestion has been implemented in the revised manuscript |
| Lines 287-292: Preferably change to: " There was maldistribution of health professionals, especially medical specialists, across the counties. This was basically due to absence of any joint formal recruitment strategy and lack of cooperation for sharing health professionals between counties. Participants suggested a coordinated sector wide collaboration and sharing of resources for an improved health system efficiency. (taken from lines 302-304)" | This suggestion has been implemented in the revised manuscript |
| Lines 304-308: From this excerpt, why a smooth mechanism was difficult might need more exploration. Is there any content to further go deeper on the bottlenecks preventing sharing of resources between counties. If there, then kindly enrich this section further with the details. Probably suggest in the paper conclusion section that further study exploring factors influencing absenteeism of health workers/ professionals could be explored. | Unfortunately we do not have further details on this issue. We have flagged it as an issue that needs further research |
| Line 315: Probably Add a line here: "Moreover, there are also governance issues where the officials do not consider healthcare staff as a priority, resulting in delayed payment of salaries." (taken from lines 321-323) | This suggestion has been implemented in the revised manuscript |
| Line 325-328: Preferably modify to: "Participants also reported that inadequate accountability mechanisms for especially permanent & pensionable health professionals contributed to health system inefficiency." | This suggestion has been implemented in the revised manuscript |
| Political interests & interference: |  |
| Line 343:Consider improving “...health budgets to capital assets and infrastructure over other forms of investments such as health commodities or health workers.” to “...health budgets to tangible capital assets and infrastructure over other forms of intangible investments such as health commodities or health workers.” | This suggestion has been implemented in the revised manuscript |
| Probably add a line between 357-358: " This resulted in discouraging people from taking up the post of health manager leading to further health system inefficiencies." | This suggestion has been implemented in the revised manuscript |
| Corruption: This section is all-right. |  |
| Co-ordination between actors: |  |
| This section provides good information on the lack of coordination of actors and its implications (like duplication) leading to health system inefficiencies. Could this section be possibly further enriched using the transcribed data to understand some of the reasons for this lack of co-ordination. | We don’t have other suitable quotes. We have included this as an area for further research |
| Discussion: |  |
| Overall, it is a very well-crafted discussion. |  |
| Line 445: Preferably replace "software aspects of health systems" to "the intangible health system factors". | This suggestion has been implemented in the revised manuscript |
| Line 460-462: Kindly check "national government to county government" as the excerpts only focused on county to facility level government. | This suggestion has been implemented in the revised manuscript |
| Line 465: Probably replace 'had lost' to 'lacked'. | This suggestion has been implemented in the revised manuscript |
| Line 479-480: (in) is not clear. | We have edited out the brackets and its content |
| Line 533-549: My opinion is that these lines are more suitable in study design to justify the qualitative study approach. | These have now been moved to the study design section |
| Limitations: Consider changing the heading to study limitation. | We don’t have a heading because we have aligned with the guidelines of the journal |
| Very well written limitation section. Some considerations: Was there any selection bias with purposive sampling? How did the researchers prevent their understanding from influencing the study outcomes? | Purposive sampling will always have some selection bias (from an epidemiological standpoint). We think that selecting a diverse range of participants, providing varied views minimizes the biasing of views. We have indicated in the methods section that we used peer debriefing and member checking. We think this minimized researcher influence of the study outcomes |
| Write in line 561: "However, the availability of thick description of the study context with rich data excerpts might help in the transferability of study findings into similar care contexts. " | This suggestion has been implemented in the revised manuscript |
| Conclusion: |  |
| Needs to improve. Write few lines on what the findings indicated as the concept of efficiency and which were the sources of health system inefficiencies. | This suggestion has been implemented in the revised manuscript |
| General: Check ref. no. 7 in the references for typographic error. Just check with COREQ checklist if all items have been covered. I would suggest to include a section on researcher reflexivity and mention the relevant details there based on domain 1 of COREQ checklist. | This suggestion has been implemented in the revised manuscript |
| Reviewer #2: |  |
| My remarks on the manuscript are below: |  |
| 1. The authors should have introduced more on the concept of health system efficiency in the background or methods section. A brief on: How does this study look different from many other studies mostly looking at efficiency at the institutional level? Where do the widely discussed concepts on technical efficiency and allocative efficiency fit or stand out in this study? | We have outlined this study is different from other studies majorly because it used a qualitative approach. We have now revised the introduction to include more details on the concept of efficiency |
| 2. In the abstract conclusion: can you please add more (public health system implications) beyond methodological recommendations. | This suggestion has been implemented in the revised manuscript |
| 3. In line number 107: backgrounds were selected as detailed in Table 1” I could not find the table attached as mentioned. | Table 1 has now been included |
| 4. Could you please add or supplement the revised manuscript with the workshop or focus group discussion guidelines that you have used as the study tools? | This has now been provided as a supplementary file |
| 5. In line number 207-209 “They also highlighted the need to select outputs and outcomes that were applicable across all counties in Kenya to facilitate valid comparisons.” Beyond this line the text does not discuss about outcomes. Could you please discuss and present what outcomes were highlighted by participants? | Participants did not provide specific outcome indicators but rather highlighted the broad areas that the indicators should represent. These are presented in the section and include – intervention coverage and outcomes for infectious diseases, RMNCAH, NCDs, quality of care |
| 6. Shortening the verbatim could enhance the readability of the manuscript. Verbatims are longer. | The verbatims have now been shortened where relevant |
| 7. In the result section in subsection PFM: Only the aspect of budget execution and disbursement are presented. So the further discussion on other aspects of PFM like the role of the county on budget formulation, external security, and audit that could affect the efficiency could enrich this manuscript. | We agree with the reviewer. However, these issues were not brought out by the participants. We have now flagged this as an area for potential study |
| 8. In line number 530, Please correct the reference abiding by the Journal styles. | This has now been corrected |
| 9. The paragraph between lines number 528-521 discussed about the importance of qualitative research methods could be summarized and shortened. | This has now been shortened, and most of it moved to the methods section as suggested by reviewer 1 |
| 10. Please add and elaborate strengths of this study along with the limitations? | We have now included strengths of the study |
| 11. What are the implications of this study's findings on the public health system of Kenya? Please elaborate. | We have now included implications in the discussion section |
